# Reduction of Specific Circulating Lymphocyte Populations with Metabolic Risk Factors in Patients at Risk to Develop Type 2 Diabetes

**DOI:** 10.1371/journal.pone.0107140

**Published:** 2014-09-25

**Authors:** Helena Cucak, Dorte Vistisen, Daniel Witte, Annelotte Philipsen, Alexander Rosendahl

**Affiliations:** 1 Hagedorn Research Institute. Department of Diabetic Complications Biology Novo Nordisk A/S, Måløv, Denmark; 2 Department of Clinical Epidemiology, Steno Diabetes Center, Gentofte, Denmark; New York University, United States of America

## Abstract

Low-grade inflammation, characterized by increased pro-inflammatory cytokine levels, is present in patients with obesity-linked insulin resistance, hyperglycemia and hyperlipidemia and considered to play a leading role to progression into type 2 diabetes (T2D). In adipose tissue in obese patients and in pancreatic islets in T2D patients cellular inflammation is present. However, the systemic leukocyte compartment and the circulating endothelial/precursor compartment in patients at risk to develop T2D has so far not been analyzed in detail. To address this, peripheral blood cells from a cohort of 20 subjects at risk to develop diabetes with normal to impaired glucose tolerance were analyzed by flow cytometry using a wide range of cellular markers and correlated to known metabolic risk factors for T2D i.e. fasting plasma glucose (FPG), 2 h plasma glucose (2 h PG), HbA1c, body mass index (BMI), homeostasis model assessment of β-cell function (HOMA-B), homeostasis model assessment of insulin sensitivity (HOMA-IS) and fasting insulin (FI). The four highest ranked cell markers for each risk factor were identified by random forest analysis. In the cohort, a significant negative correlation between the number of TLR4^+^ CD4 T cells and increased FPG was demonstrated. Similarly, with increased BMI the frequency of TLR4^+^ B cells was significantly decreased, as was the frequency of IL-21R^+^ CD4 T cells. Unlinked to metabolic risk factors, the frequency of regulatory T cells was reduced and TLR4^+^ CD4 T cells were increased with age. Taken together, in this small cohort of subjects at risk to develop T2D, a modulation of the circulating immune cell pool was demonstrated to correlate with risk factors like FPG and BMI. This may provide novel insights into the inflammatory mechanisms involved in the progression to diabetes in subjects at risk.

## Introduction

The prevalence of obesity and severe obesity has increased to epidemic levels during the last decades with recent numbers indicating that close to 20% of the adult population on a global scale does have a BMI above 30 [1]. The worldwide increase in overweight and obesity has been closely evaluated and shown to be strongly associated with an increase in the prevalence of T2D [2], [3]. Currently, close to 300 million people have T2D and the number is predicted to increase to approximately 450 million by 2030 [4]. Among people with pre-diabetes, the incidence of onset of T2D is reduced by approximately 40 to 45% with effective lifestyle changes or drug treatment and up to almost 80% with bariatric surgery [5]. For the patients that progress into T2D, a significant risk of developing associated diabetes complications like diabetic nephropathy, stroke and macro vascular diseases like atherosclerosis exists [6], [7]. For this reason, early identification of patients with undiagnosed T2D or careful evaluation of obese or normal weight patients displaying an increased risk of developing T2D remains an important clinical challenge.

Over the last decade, an abundance of evidence has demonstrated a close link between the immune system and obesity [8]. It is firmly established that a chronic low grade inflammation exists in obese patients and animal models of obesity which is instrumental for development of insulin resistance [9]. The low grade inflammation is characterized by increased levels of circulating inflammatory cytokines and chemokines like IL-1β, IL-6, TNFα and MCP-1. Animal models have demonstrated that the vast majority of these inflammatory mediators are derived from the adipose tissue and the liver [8]. In the adipose tissue an intricate interaction between the few resident leukocytes and the adipocytes creates a local milieu favoring a pro-inflammatory signature of cytokines which attracts peripheral blood monocytes and lymphocytes towards the adipose tissue [10], [11]. Once there, the monocytes polarize towards M1-like macrophages and contribute to fueling the local inflammation further. With progression the local adipose environment is altered and cytokines promoting tissue scarring like TGF-β are produced [12]. At this stage the low grade circulating inflammatory mediators have resulted in an amplified white blood cell count (WBC) and altered the activity state of some immune cells in obese individuals [13]. Indeed, elevated WBC has been shown to predict the development of insulin resistance and to correlate to reduced insulin secretion in obese patients [14].

Evidence now suggests T2D to be characterized as an inflammatory disease [8]. The vast majority of the T2D diabetic patients are overweight to obese and do show obesity linked insulin resistance, hyperglycemia, hyperlipidemia and elevated inflammatory circulating factors [9]. Elevated levels of acute-phase proteins (e.g. CRP) as well as several cytokines (e.g. IL-1β, IL-6) are present in T2D patients and are shown to be modulated during disease progression [15]. Further, in the diabetic islets of Langerhans, an accumulation of primarily M1-like monocytes takes place already when the disease is developing [16]. However, in established diabetes splenic macrophages are altered towards a phenotype more resembling alternatively activated macrophages expressing CD206 and galectin-3 associated with high production and TGF-β signaling capacity [16].

In T1D, the circulating T cells express the activated CD3^+^DR^+^CD30^+^ phenotype [17]. Further, in T1D peripheral blood there is an enhanced number of IL-21 receptor expressing Tfh cells and these posses an enhanced capacity to produce IL-21 upon re-challenge [18]. IL-21R positive T cells have been shown to play non-redundant roles in several systemic inflammatory conditions like rheumatoid arthritis [19], [20]. Toll like receptor (TLR)-4 is classically associated with myeloid cells and works as signaling receptor through which the cells sense bacterial infections [21]. Recently, expression and activation of TLR4 on various lymphoid cells e.g. CD4 T cells was demonstrated where it has been suggested to act as a negative amplification of the immune responses limiting the excessive inflammation [22], [23].

To address if an altered leukocyte population could be identified in peripheral blood in patients at risk to develop T2D, various inflammatory cell phenotypes were evaluated in a small cohort. The cellular phenotypes were correlated to known risk factors for development of T2D before and after food intake to monitor if intake of normal diet influences the peripheral blood immune cell composition. Our results demonstrate that already in subjects at risk to develop T2D, modulation of the peripheral immune cell composition in the peripheral blood could be detected. The changes included reduction of TLR4^+^ T cells with increased FPG and TLR4^+^ B cells as well as IL-21R^+^ T cells with increased BMI. Thus, the data in this small cohort provide novel insights to the systemic low grade inflammation occurring in patients at risk to develop T2D potentially providing enhanced mechanistic understanding of the disease progression.

## Materials and Methods

### Study population

Participants were recruited from the ADDITION-PRO study, a follow-up health examination in 2009–2011 of a Caucasian cohort of participants at high risk of developing T2D identified through stepwise screening in Danish general practice. Details of this population have been described elsewhere [24]. At baseline in 2001–2006, the 20 participants examined here were at high risk of developing T2D based on a diabetes risk score questionnaire, however had normal glucose tolerance.

In the ADDITION-PRO study an oral glucose tolerance test was performed providing data on FPG and 2 h PG. Further, the information on HbA1c, BMI, HOMA-B, HOMA-IS and FI was also obtained at the ADDITION-PRO examination. On a separate examination day 14,4 months later, blood samples were taken upon a fasted state of at least 8 hours and 75 minutes after a meal for the analysis of immune cell markers. The participants were given 15 minutes to eat and drink as much as possible of a meal consisting of three sandwiches, a pear, water, juice, tea and coffee. The sandwiches consisted of 14% protein, 34% fat and 52% carbohydrates, reflecting an average Danish diet. Amounts consumed were recorded.

### Ethical permission

The study was approved by the scientific ethics committee of Central Denmark Region (approval number M-20080229) and performed in accordance with the declaration of Helsinki. All participants gave written informed consent.

### Collection of patient blood

10 ml total peripheral blood in EDTA was received from 20 individuals with different glycemic stages before and after a meal. Red blood cells were lysed in RBC lysis buffer (cat no: 420301 Biolgend) according to the manufacturer’s protocol.

### Flow cytometry analysis

Flow cytometric analysis was performed according to standard procedures. Briefly, cells were first blocked for unspecific binding with anti-CD16 (NKP15, BD Biosciences) and anti-CD32 (FL18.26, BD Pharmingen) followed by surface staining of CD11c (BU15, Biolegend), CD163 (GHI/61, Biolegend), TLR4 (HTA125, Biolegend), CXCR5 (RF8B2, BD), CD3 (SK7, eBioscience), CD4 (OKT4, Biolegend), ICOS (ISA-3, eBioscience), CD19 (HIB19, eBioscience) 7-amino-actinomycin D (7-AAD) (Biolgend), CD25 (BC96, Biolegend), CD127 (A019D5, Biolegend), CTLA4 (L3D10, Biolegend), IL-21R (17A12, BD Pharmingen), CD31 (WM59, Biolegend), CD34 (581, Biolegend), CD45 (HI30, Biolegend) and CD133/1 (AC133, Miltenyi Biotec). Cells were then fixed and permeabilized using Cytofix/Cytoperm Fixation/Permeabilization Solution Kit (BD Pharmingen) according to manufacturer’s description and then intracellularly stained for CD68 (Y1/82A, Biolegend) and FoxP3 (206D, Biolegend).

Intracellular cytokine levels were evaluated in peripheral blood cells cultured in standard media (RPMI+10%FCS and P/S, Sigma) at 1.6*10^6^/ml in the presence of 50 ng/ml PMA and 1 µg/ml ionomycin. After 1 hour, 10 µg/ml BrefeldinA was added to the cultures and the cells were incubated for 4 additional hours. Cells were stained for surface antigens i.e. CD4, CD3 and CD19 and followed by fixation and permeabilization and finally stained for IL-4 (8D4-8, BD Pharmingen), IFN-γ (B27, BD Pharmingen), IL-17 (eBio64DEC17, eBioscience) and IL-21 (3A3-N2, Biolegend).

All samples were acquired on a FACSFortessa equipped with blue, red and violet laser followed by data analysis using FACSdiva software (BD Biosciences).

### Statistical analysis

The information on FPG, 2 h PG, HbA1c, BMI, HOMA-B, HOMA-IS and FI obtained at the ADDITION-PRO examination 14.4 (SD: 0.4) months before, was used in order to conducted the statistical evaluations. At the time of blood sampling for the analysis of peripheral blood inflammatory cell markers, participants did not receive an OGTT. For some individuals BMI, FPG and HbA1c was collected at both examination occasions and the derived range was small and within the range of intra-person variability (median difference (IQR): BMI: 0.22 kg/m^2^ (−0.13;0.45); FPG: −0.35 mmol/l (−0.60; −0.05); HbA1c: 0.00% (−0.10;0.10)).

Given the high number of cellular immune markers to the number of participants in our study, an initial screening of the markers employing Random Forests analysis to FPG, 2 h PG, HbA1c, BMI, HOMA-B, HOMA-IS, FI, age and sex was conducted [25]. In brief, the Random Forests algorithm constructs an ensemble of classification or regression trees (10.000 trees in this study) from several bootstrap samples of the original data and votes or averages over the trees in order to increase prediction [26]. Bootstrapping is sampling with replacement, and in each bootstrap, about one-third of the study participants are left out of the construction of a particular tree. This so-called out-of-bag sample is used as test data for calculating the error rate of the derived classification tree. For each immune phenotype included in the analysis, the Random Forests algorithm computes an estimate of the increase in error rate of the classification/regression tree had that marker not been used, a procedure named permutation test. The permutation test was used to rank the cell markers, and the four highest ranking markers were selected for further analysis. Subsequently, associations between cell markers and the studied outcomes were assessed in linear- or logistic regression analysis adjusting for age and sex. In the analyses of age and sex as outcomes, adjustment was only made for the other, i.e. age or sex. A level of significance of 5% was adjusted for multiple testing (n = 36) using the method by Benjamini et al. [27] Random forest analysis is illustrated in Fig. S1.

## Results

### Cohort description

Of the 20 participants studied, 60% were men, they had a mean age of 68.7 years (SD: 6.4) ranging from 53 to 78 years. Mean BMI was 27.2 kg/m^2^ (SD: 4.1), FPG was 5.6 mmol/l (SD: 0.6), 2 h PG 5.6 mmol/l (SD: 1.4), mean HbA1c was 5.7% (SD: 0.4), FI ranged between 11–109 pmol/ml, HOMA-B ranged between 22–120.7% and HOMA-IS 0.24–3.23%. Four of the study participants had pre-diabetes (all men), while the rest had normal blood glucose but were classified as being at high risk to develop diabetes (Table 1) [24].

**Table 1:** Characteristics of the investigated ADDITION-PRO sub-cohort.

Participants (n)	20
Age (years)	53–78
Gender (% female)	40
Fasting plasma glucose(mmol/l)	4.4–6.7
2-hour plasma glucose(mmol/l)	3.3–10.3
HbA1c (%)	5.1–7.1
Body mass index (kg/m^2^)	21.2–36.5
Fasting insulin (ρmol/l)	11–109
HOMA-B (%)	22.0–120.7
HOMA-IS	0.24–3.23

### The frequency of monocyte subsets was not changed upon food intake

Elevated WBC has been demonstrated in obese patients, but the identity of the cells is not yet fully understood [13]. Food intake significantly changes the composition of WBC in healthy individuals as early as 1 h post a light meal [28]. To determine which monocyte sub-populations where present in subjects at risk to develop T2D and if these were changed after food intake, a flow cytometric analysis of peripheral blood from the cohort was conducted.

**Figure 1:**
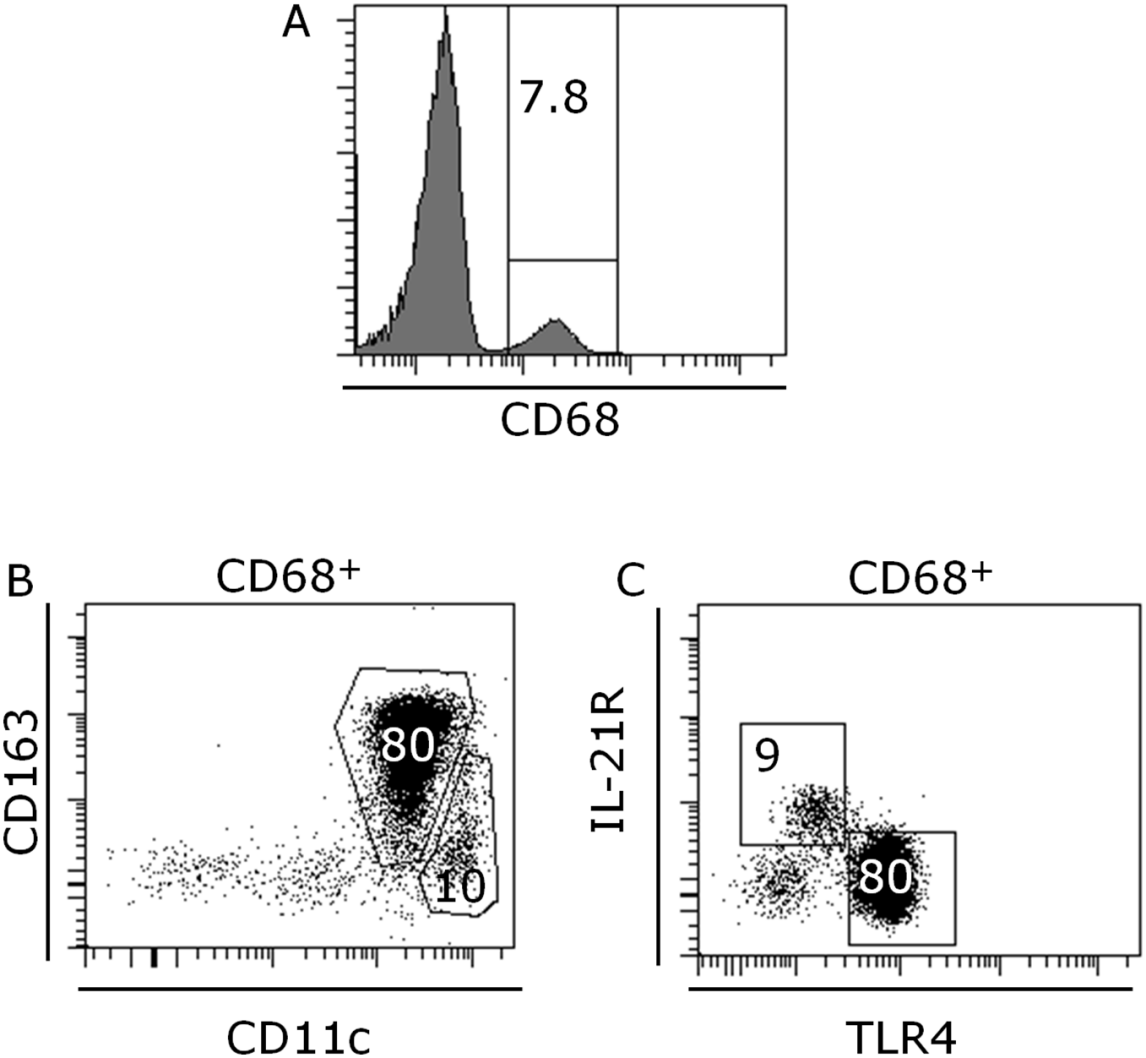
Representative flow cytometric analysis of peripheral blood CD68 positive monocytes (A), M1-like CD163^−/int^CD11c^high^ monocytes and M2-like CD163^int/hi^CD11c^int^ monocytes (B), IL-21R^+^ monocytes and TLR4^+^ monocytes (C). The numbers represent percentage of cells within the gates. At least 10^6^ total cells were acquired followed by gating on size versus granularity followed by exclusion of dead cells and finally detection of markers described in plots.

The data showed that 7.8% of the peripheral white blood cells were CD68 positive monocytes in the cohort (Table 2, Fig. 1A). The predominant subset of these monocytes (80%) were CD163^int/hi^CD11c^int^ M2-like cells while only 10% of the CD68^+^ cells represented CD163^−/int^CD11c^high^ M1-like subset (10%) (Table 2, Fig. 1B). Approximately 9% of the monocytes were IL-21R^+^ rendering them responsive to IL-21 (Table 2, Fig. 1C). 80% of the monocytes expressed TLR4 and thus were responsive to danger signals from bacterial infection derived LPS as well as from endogenously derived stress signals like HMGB1 and hsp. Importantly, none of the monocyte sub-populations were significantly changed after food intake (Table 2).

**Table 2:** Monocytes in peripheral blood. Data is displayed as mean ± standard deviation.

			Condition		
			Before meal	After meal	Before meal
	Cell type	Specific marker(s)	(%)	(%)	(total number)
of tot blood	Monocyte	CD68^+^	7.790±1.523	7.292±1.25	460854±217066
		CD68^+^IL21R^+^TLR4^−^	9.115±4.394	8.086±3.98	38643±22879
	Monocyte				
of CD68^+^		CD68^+^IL21R^−^TLR4^+^	80.23±6.734	79.2±6.443	374413±203795
	M1 like monocyte	CD68^+^CD163^−/int^CD11c^high^	10.4±5.024	10.60±5.26	43718±24462
	M2 like monocyte	CD68^+^CD163^int/hi^CD11c^int^	79.66±6.487	78.29±8.263	373838±208364

Taken together, the data shows that neither the monocytes nor the sub-populations of monocytes were modulated by food intake.

### Food intake rapidly expanded the TLR4^+^ T cells, while B cell subsets were unaffected

Modulation of T and B cells in T1D has been observed, but the impact on T cell and B cell subsets in patients at risk for T2D has not been established yet [17]. As food intake has been demonstrated to modulate the composition in healthy individuals as early as 1 h post a light meal, the number of T and B cell sub-populations in subjects at risk to develop T2D was determined both pre and post food intake by flow cytometric analysis [28]. Further, to identify the cytokine signature of the blood cells an *in vitro* re-challenge was performed.

**Figure 2:**
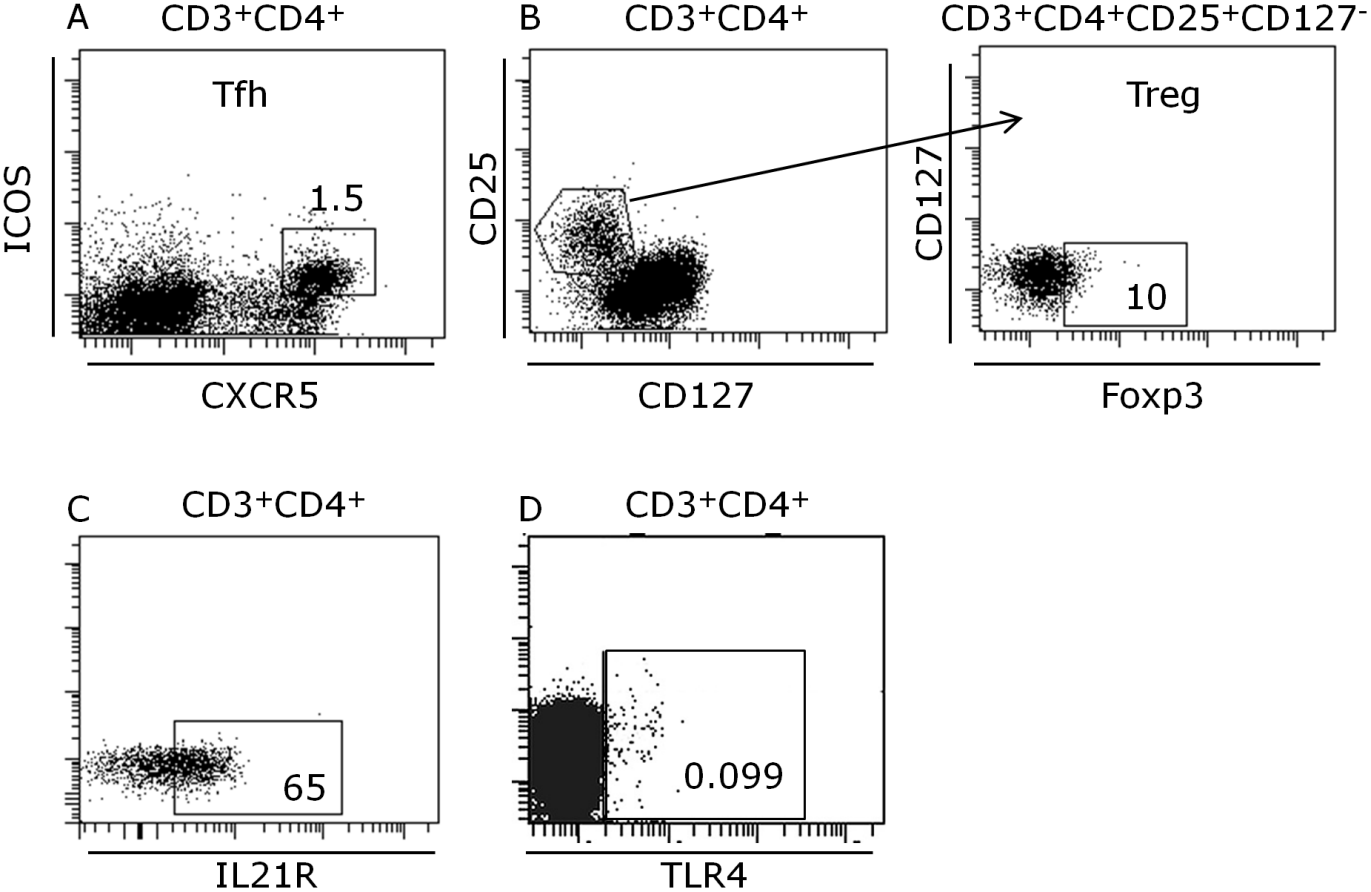
Representative flow cytometric analysis of peripheral blood CD3^+^CD4^+^CXCR5^+^ICOS^+^ Tfh cells (A), CD3^+^CD4^+^CD25^+^CD127^−^Foxp3^+^ Treg cells (B), CD3^+^CD4^+^IL-21R^+^ T cells (C) and CD3^+^CD4^+^TLR4^+^ T cells (D). The numbers represent percentage of cells within the gates. At least 10^6^ total cells were acquired followed by gating on size versus granularity followed by exclusion of dead cells and finally detection of markers described in plots.

**Figure 3:**
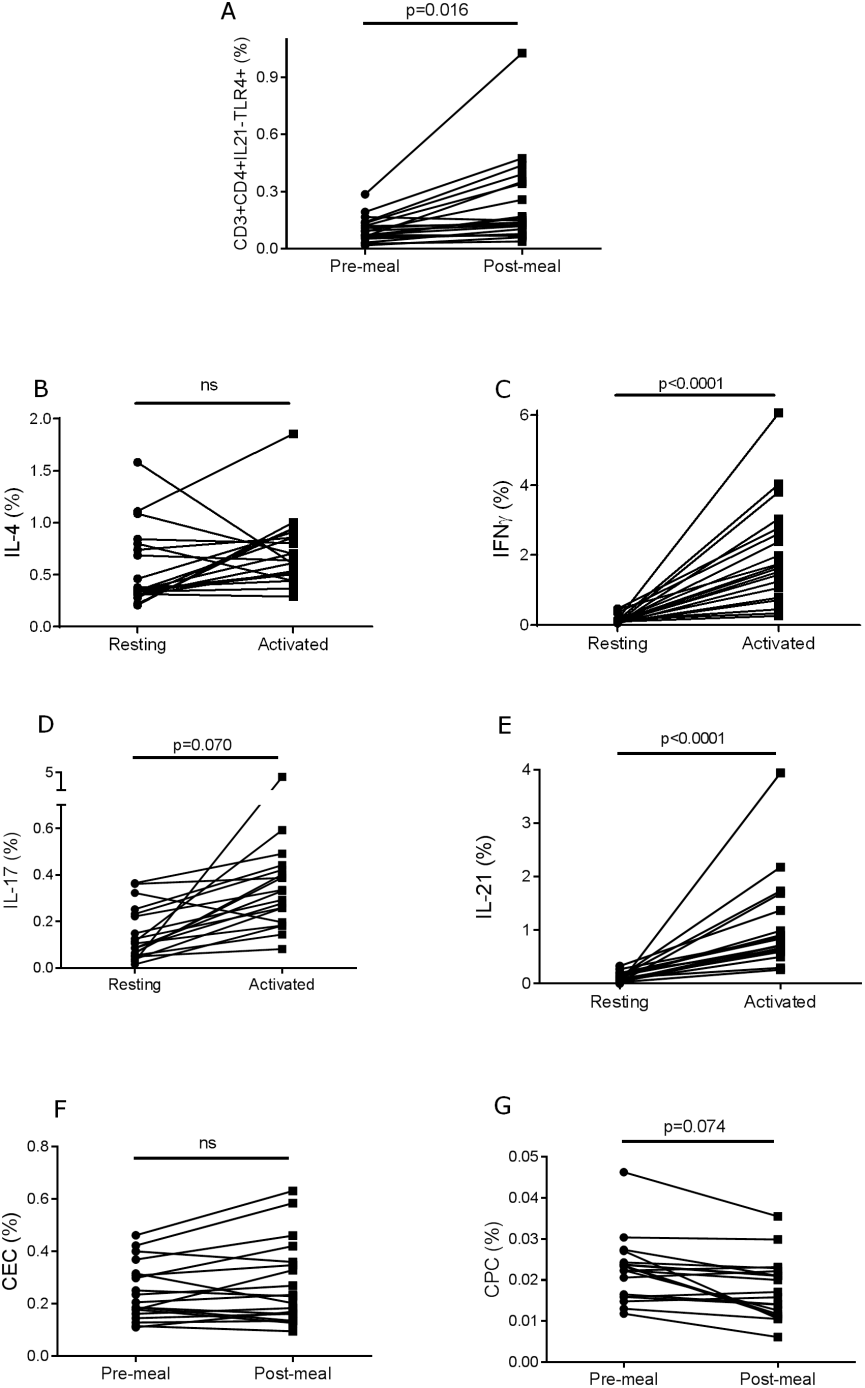
Frequency of TLR4^+^ T cells in peripheral blood before and 75 min post meal (A). Cytokine production in CD4^+^ T cells in peripheral blood before and after activation of the cells for 4 h *ex vivo*: IL-4 (B), IFN-γ (C), IL-17 (D), IL-21 (E). Frequency of CECs (F) and CPCs (G) in peripheral blood before and 75 min post meal. Statistics was obtained using unpaired two-way T-test using Welsh correction.

Approximately 8% of the peripheral white blood cells were CD4 positive T cells (Table 3). Out of these, 1.5% was T follicular helper cells (Tfh) (Table 3, Fig. 2A). 0.6% of the peripheral white blood cells were CD3^+^CD4^+^CD25^+^CD127^−^ previously shown to have Treg cell phenotype [29] (Table 3). To confirm that these were the true Tregs we stained them for Foxp3. Unexpectedly out of these cells only 10% expressed FoxP3 (Fig. 2B). Since Liu *et al.* [29] have previously shown that majority of CD3^+^CD4^+^CD25^+^CD127^−^ cells express Foxp3 we suspect insufficient permeabilization of our samples why we decided to define Tregs as CD3^+^CD4^+^CD25^+^CD127^−^ T cells without including Foxp3. 65% of the CD4^+^ T cells were IL-21R^+^ (Table 3, Fig. 2C). None of these CD4 populations were changed after food intake (Table 3). A small population of CD4^+^ T cells (0.099%) expressed the innate receptor TLR4 before meal (Fig. 2D). In contrast, only 75 minutes after food intake a significantly (p = 0.016) 2.5-fold increase of this TLR4^+^ population to 0.23% of the CD4^+^ T cells was demonstrated (Table 3, Fig. 3A).

**Table 3:** Peripheral blood T cells. Data is displayed as mean ± standard deviation.

			Condition		
			Before meal	After meal	Before meal
	Cell type	Specific marker(s)	(%)	(%)	(total number)
	T cell	CD3^+^CD4^+^	8.185±2.958	7.546±2.992	455821±184942
of total blood					
	T reg	CD3^+^CD4^+^CD25^+^CD127^−^	0.6463±0.2083	0.6346±0.16	37674±17187
		CD3^+^CD4^+^IL21R^+^TLR4^−^	64.78±10.21	66.44±7.236	304637±151921
	T cell				
of CD3^+^CD4^+^		CD3^+^CD4^+^IL21R^−^TLR4^+^	0.09939±0.0635	0.2341±0.22	393.7±192.6
	Tfh like cell	CD3^+^CD4^+^CXCR5^+^	11.61±2.956	11.21±3.268	51971±23530
	Tfh	CD3^+^CD4^+^CXCR5^+^ICOS^+^	1.464±1.297	1.281±1.249	5776±4115
		IL-4	0.7274±0.3377	N.A.#	2996±1095
of activated	T cell	IFNγ	1.977±1.445	N.A.	8794±5949
CD3^+^CD4^+^		IL-17	0.485±0.8110	N.A.	1738±1477
		IL-21	1.054±0.8364	N.A.	4205±2762
		IL-4	0.5528±0.3692	N.A.	2190±1117
of resting	T cell	IFNγ	0.1452±0.1239	N.A.	594±467.1
CD3^+^CD4^+^		IL-17	0.1825±0.2043	N.A.	815.8±757.7
		IL-21	0.1142±0.08600	N.A.	524.5±449.5

# Not analyzed.

Basal production of the cytokines IL-4, IL-21, IFN-γ and IL-17 was present in the T cells (Table 3). Importantly, activation of the cells for only 4 h *ex vivo* induced strong upregulation particularly of IFN-γ (p<0.0001) and IL-21 (p<0.0001) and partly of IL-17 (p = 0.07), while IL-4 was not induced suggesting that the peripheral cells showed a Th1/Th17/Tfh polarized cytokine signature (Table 3, Fig. 3B–E).

**Figure 4:**
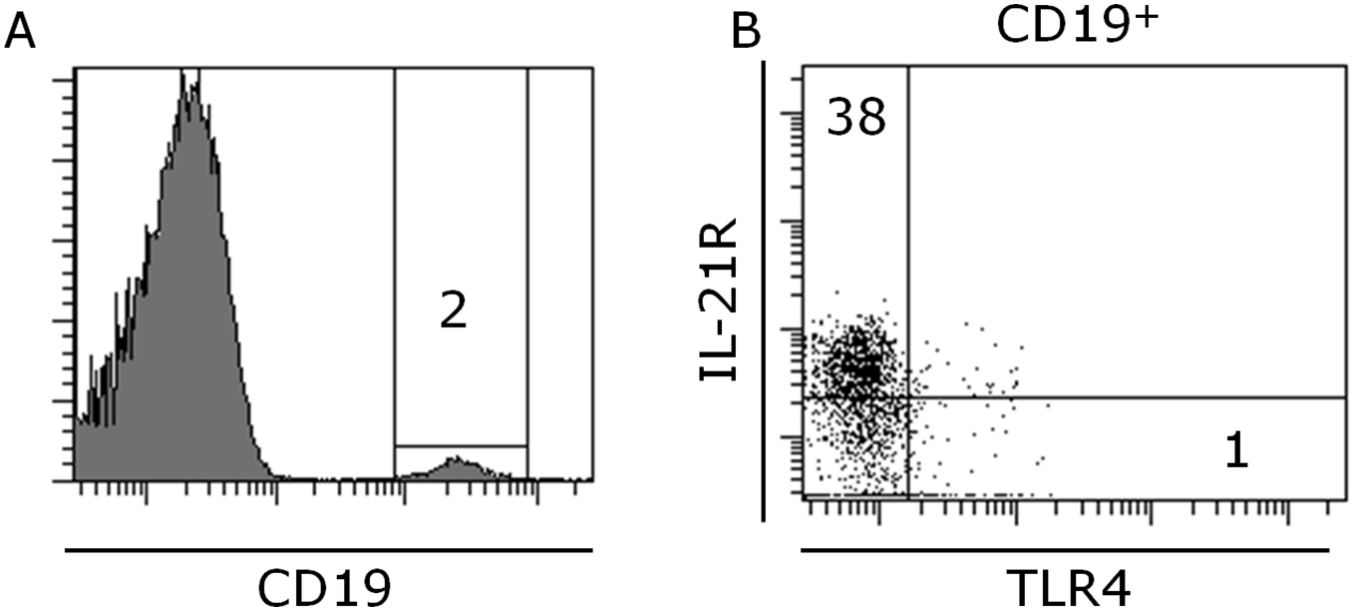
Representative flow cytometric analysis of peripheral blood CD19 positive B cells (A), IL-21R versus TLR4 on B cells (B). The numbers represent percentage of cells within the gates/quadrants. At least 10^6^ total cells were acquired followed by gating on size versus granularity followed by exclusion of dead cells and finally detection of markers described in plots.

The frequency of B cells in blood was 2%, of which 38% were IL-21R^+^, while only 1% expressed the TLR4 (Table 4, Fig. 4A–B). The B cells and the B cell subsets were not affected by food intake.

**Table 4:** Peripheral blood B cells. Data is displayed as mean ± standard deviation.

			Condition		
			Before meal	After meal	Before meal
	Cell type	Specific marker(s)	(%)	(%)	(total number)
of tot blood	B cell	CD19^+^	2.092±0.8595	2.031±0.8952	131638±117560
of CD19^+^		CD19^+^IL21R^+^TLR4^−^	38.43±12.45	41.08±14.17	43258±25736
		CD19^+^IL21R^−^TLR4^+^	1.069±1.060	1.258±0.9168	1301±1749

Taken together, food intake rapidly influenced the small TLR4^+^ subset of the CD4^+^ compartment in peripheral blood, while other subsets and the B cell compartment remained unaffected by food intake.

### Food intake showed a trend towards reduction of circulating endothelial precursor cell numbers

Vasculature stress and inflammation to the endothelium induces repair mechanisms involving circulating endothelial cells (CECs) and the circulating endothelial precursor cells (CPCs) [30], [31]. To address if the frequency of these cells was changed upon food intake in the cohort of subjects at risk to develop T2D, a flow cytometric analysis of the CPCs and CECs was performed.

**Figure 5:**
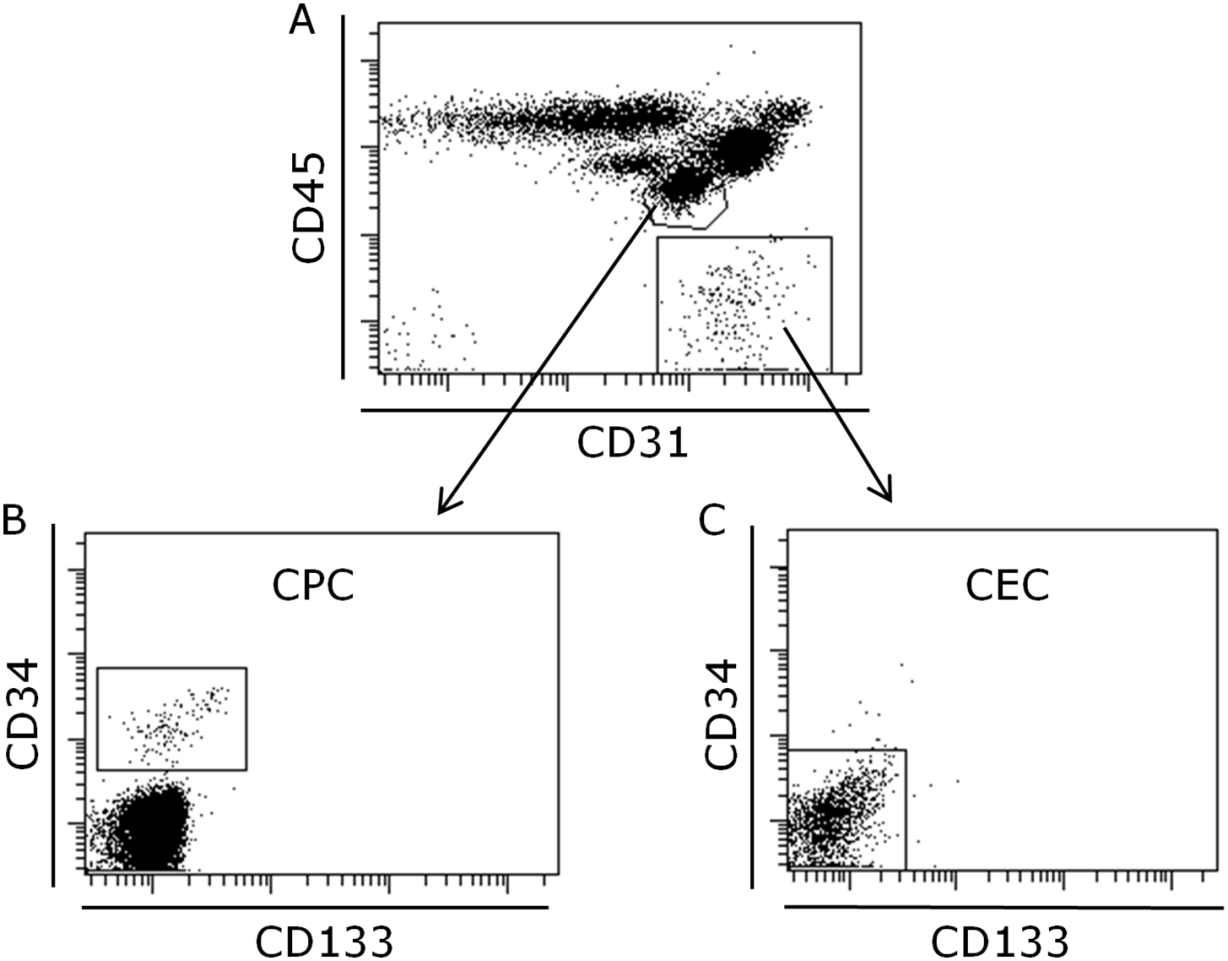
Representative flow cytometric analysis of peripheral blood, CD45 versus CD31 (A), CD31^+^CD34^+^CD45^dim^CD133^dim^ CPC cells (B) and CD31^bright^CD34^−^CD45^−^CD133^−^ CEC cells (C). The numbers represent percentage of cells within the gates. At least 10^6^ total cells were acquired followed by gating on size versus granularity followed by exclusion of dead cells and finally detection of markers described in plots.

The CECs were identified as cells expressing very high levels of the endothelial marker CD31 and being negative for CD34, CD133 and CD45 (Fig. 5A and C). The frequency of these cells was 0.26% of the peripheral blood cells and was not changed upon food intake (Table 5, Fig. 3F). The CPCs were identified by expression of the stem cell marker CD34 together with CD45, CD31 and CD133 (Fig. 5A and B). The frequency of these cells was 0.02% of the peripheral blood (Table 5). Noteworthy, there was a non-significant trend to a decrease after food intake (Fig. 3G).

**Table 5:** Circulating endothelial and endothelial precursor cells.

			Conditions		
			Before meal	After meal	Before meal
	Cell type	Specific marker(s)	(%)	(%)	(total number)
	CEC	CD31^bright^CD34^−^CD45^−^CD133^−^	0.2639±0.1357	0.2798±0.1536	15258±8610
of tot blood					
	CPC	CD31^+^CD34^+^CD45^dim^CD133^dim^	0.02257±0.007553	0.01853±0.007578	1262±424

Data is displayed as mean ± standard deviation.

Thus, the data demonstrates that food intake does not alter the presence of CECs, while a trend to a decreased frequency of CPCs was noted 75 minutes post meal.

### Identification of a modulated peripheral blood immune cell composition in patients at risk for T2D

To determine if the peripheral blood immune sub-populations in the cohort of subjects at risk to develop T2D showed a statistical correlation to metabolic risk factors such as FPG, 2 h PG, HbA1c, BMI, HOMA-B, HOMA-IS and FI a correlation analysis was performed and adjusted for age and sex. The adjustment for age and sex was performed in order to avoid misinterpretation of the data since all pre-diabetic individuals in our study were male and some subsets of immune cells tend to change with age [32] and could differ in number in male compared to female [33]. To identify the four highest ranked cell markers, a random forest analysis for each metabolic risk factor and age and sex was made separately. The four highest ranking markers were further analyzed by linear/logistic regression analysis Fig. S1.

### TLR4^+^ CD4 T cells were negatively correlated with increased fasting plasma glucose

**Figure 6:**
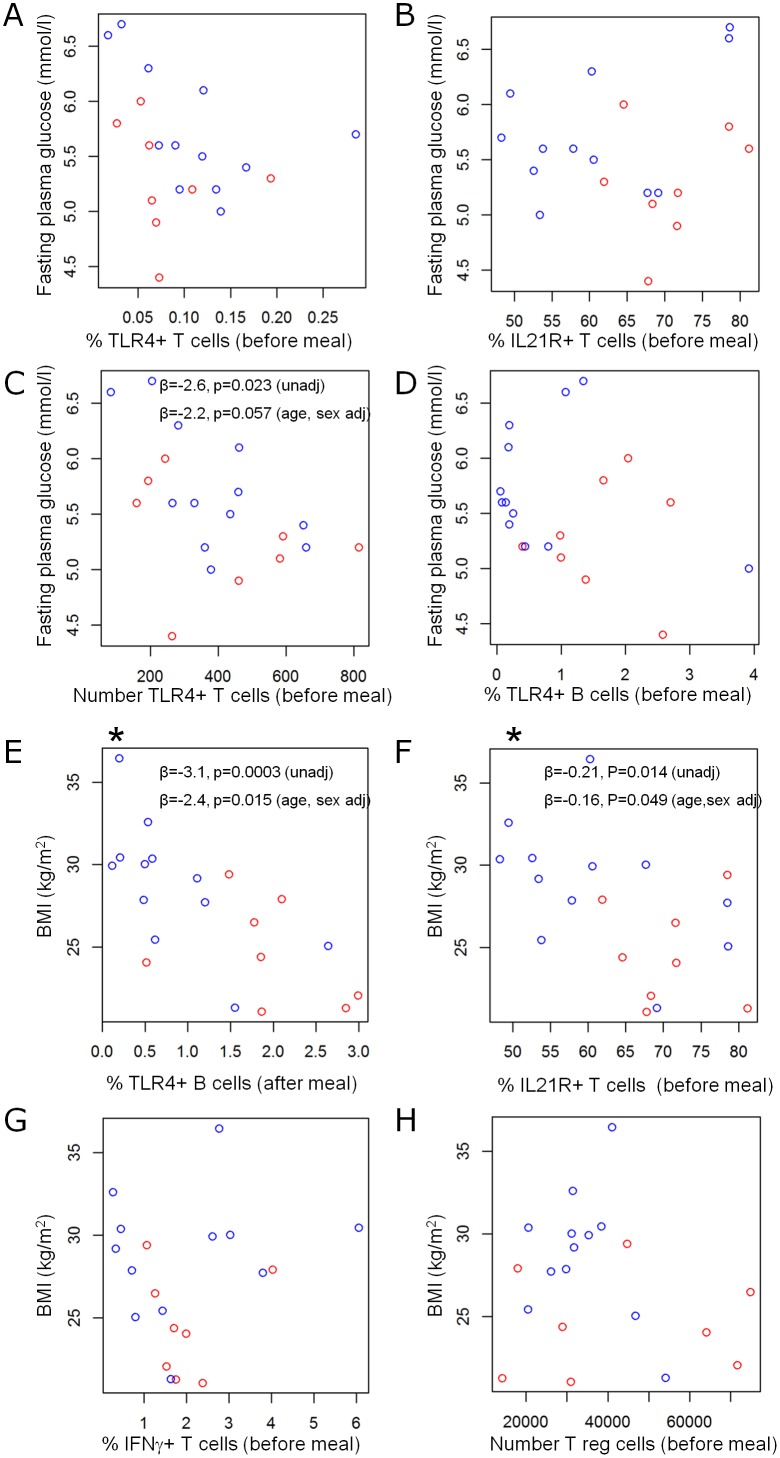
FPG (mmol/l) vs before meal frequency of CD3^+^CD4^+^IL21R^−^TLR4^+^ T cells (A), before meal frequency of CD3^+^CD4^+^IL21R^+^TLR4^−^ T cells (B), before meal number of CD3^+^CD4^+^IL21R^−^TLR4^+^ T cells (C) and before meal frequency of CD19^+^IL21R^−^TLR4^+^ B cells (D). BMI vs after meal frequency of CD19^+^IL21R^−^TLR4^+^ B cells (* = significant after adjustment for multiple testing) (E), before meal frequency of CD3^+^CD4^+^IL21R^+^TLR4^−^ T cells (F), before meal frequency of activated IFN-γ^+^ T cells (G) and before meal number of CD3^+^CD4^+^CD25^+^CD127^−^ T cells (H). Red dots represent women and blue dots represent men.

The four highest ranking markers identified when evaluated against FPG were CD3^+^CD4^+^IL-21R^−^TLR4^+^ (%) before meal, CD3^+^CD4^+^IL-21R^+^TLR4^−^ (%) before meal, CD3^+^CD4^+^IL-21R^−^TLR4^+^ (number) before meal and CD19^+^IL-21R^−^TLR4^+^ (%) before meal (Fig. 6A–D). Most interestingly, a negative correlation between the number of CD3^+^CD4^+^IL21R^−^TLR4^+^ T cells and FPG before meal (p = 0.023) was obtained (Fig. 6C). When adjusted for age and sex, the level of significance was reduced reaching the border line (p = 0.057) in the small cohort, but still showing a clear trend. No significance was observed with the other three highest ranked cell subsets (Fig. 6A, B, D).

These data suggest that the number of TLR4^+^ CD4 T cells is reduced in subjects at risk for T2D with an increase in FPG.

### TLR4^+^ B cells and IL-21R^+^ CD4 T cells were negatively correlated with BMI

The four highest ranking markers identified when evaluated against BMI were CD19^+^IL-21R^−^TLR4^+^ (%) after meal, CD3^+^CD4^+^IL-21R^+^TLR4^−^ (%) before meal, CD3^+^CD4^+^IFN-γ^+^ (%) before meal and CD3^+^CD4^+^CD25^+^CD127^−^ (number) before meal (Fig. 6E–H). Importantly, a highly significant (p = 0.0003) reduction of CD19^+^IL21R^−^TLR4^+^ B cells after food intake was observed with increasing BMI in the subjects. The reduction remained after adjustment for both age and sex (p = 0.015) as well as after adjustment for multiple testing (indicated by *) (Fig. 6E). Interestingly, the frequency of CD3^+^CD4^+^IL21R^+^TLR4^−^ T cell before meal was significantly (p = 0.014) negatively correlated to an increase in BMI (Fig. 6F). This reduction of IL-21R^+^ T cells remained after adjustment for age and sex (p = 0.049). There was no significant correlation with the other two highest ranking immune markers (Fig. 6G and H).

These data suggest that an increase in BMI in subjects at risk for T2D was correlated with a reduction in the frequency of TLR4^+^ B cells and IL-21R^+^ CD4 T cells.

### No immune cell phenotype showed an association to 2 h PG, HbA1c, HOMA-B, HOMA-IS and FI

Two-hour plasma glucose, HbA1c, HOMA-B, HOMA-IS and FI did not show any significant correlation with any cell markers tested (data not shown and Fig. S2, S3).

### Tregs were reduced, while TLR4^+^ CD4 T cells were enhanced with age

**Figure 7:**
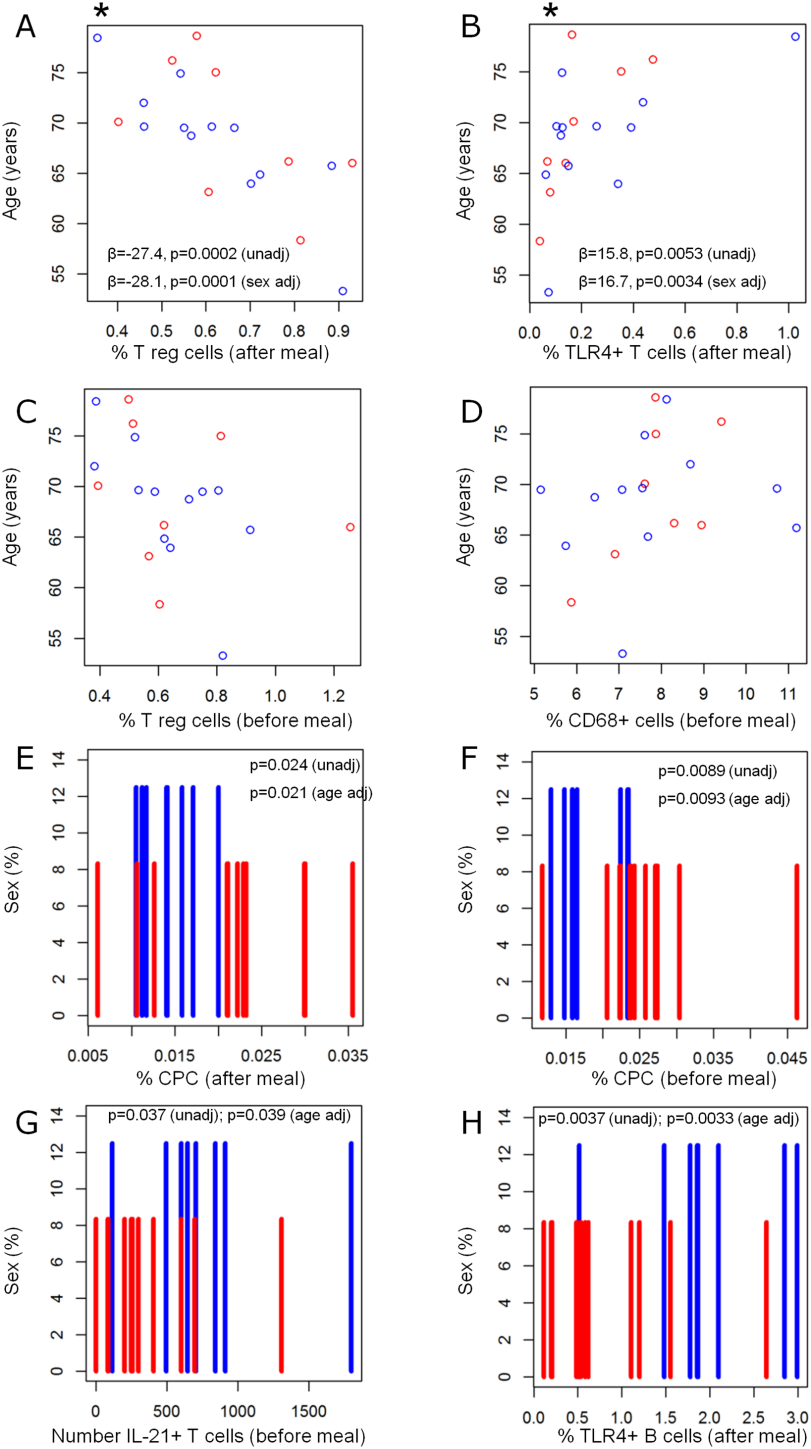
Age (years) vs after meal frequency of CD3^+^CD4^+^CD25^+^CD127^−^ T cells (A), after meal frequency of CD3^+^CD4^+^IL21R^−^TLR4^+^ T cells (B), before meal frequency of CD3^+^CD4^+^CD25^+^CD127^−^ T cells (C) and before meal frequency of CD68^+^ macrophages (D). Red dots represent women and blue dots represent men. Distribution of men and women separately vs after meal frequency of CD31^+^CD34^+^CD45^dim^CD133^dim^ CPCs (E), before meal frequency of CD31^+^CD34^+^CD45^dim^CD133^dim^ CPCs (F), before meal number of resting IL-21^+^ T cells (G) and after meal frequency of CD19^+^IL21R^−^TLR4^+^ B cells (H). Red bars represent women and blue dots represent men. (A and B * = significant after adjustment for multiple testing).

Although not a metabolic risk factor, the incidence of T2D increases with age [34]. This has not yet been evaluated in subjects at risk to develop T2D and hence this study monitored the correlation between age and peripheral blood immune sub-populations in this cohort. The four highest ranking markers identified when evaluated against age were CD3^+^CD4^+^CD25^+^CD127^−^ (%) after meal, CD3^+^CD4^+^IL-21R^−^TLR4^+^ (%) after meal, CD3^+^CD4^+^CD25^+^CD127^−^ (%) before meal and CD68 (%) before meal (Fig. 7A–D). A highly significant (p = 0.0002) decrease in frequency of CD3^+^CD4^+^CD25^+^CD127^−^ after meal was correlated with increase of age (Fig. 7A). This was further strengthened when corrected for sex (p = 0.0001) and remained significant after adjustment for multiple testing (indicated by *). Moreover, a highly significant (p = 0.0053) increase of CD3^+^CD4^+^IL21R^−^TLR4^+^ T cells was demonstrated with increased age (Fig. 7B). This increase of TLR4^+^ T cells remained when corrected for sex (p = 0.0034) and after multiple testing adjustment (indicated by *). No significance was observed with the other two highest ranked cell subsets (Fig. 7C and D).

These data suggest that the frequency of Tregs was reduced, while the frequency of TLR4^+^ CD4 T cells was enhanced with age in subjects at risk to develop T2D.

### Frequency of CPC was higher in females whereas TLR4^+^ B cells and IL-21 producing T cells were higher in males

The four highest ranking markers identified when evaluated against sex were CPC (%) after meal, CPC (%) before meal, CD3^+^CD4^+^IL-21^+^ (number) before meal, CD19^+^IL-21R^−^TLR4^+^ (%) after meal (Fig. 7E–H). The frequency of CPC after and before food intake was significantly (p = 0.024 and p = 0.0089 respectively) higher in females than in men (Fig. 7E and F). The difference remained also after age adjustment (p = 0.021 and p = 0.0093 respectively). In contrast, the capacity of CD4^+^ T cells to produce IL-21 was significantly higher (p = 0.037) in men in an age independent manner (p = 0.039) (Fig. 7G). Similarly, the frequency of CD19^+^IL-21R^−^TLR4^+^ B cells was significantly higher (p = 0.0037) in men compared to women independent of age (p = 0.0033) (Fig. 7 H).

These data showed that cell populations associated with vascular repair were more common in females, while some lymphocyte populations were more frequent in males at risk to develop T2D.

## Discussion

Obesity, familial diabetes, and higher than normal blood glucose levels increase the risk ∼25-fold to develop T2D in the next few years relative to a patient without these characteristics [35]. Due to the complexity of T2D risk physiology, several novel risk factors have emerged the last few years [36]. Among these, elevated levels of IL-6 and CRP have been significantly associated with an increased risk of T2D [37]. Inhibition of key pro-inflammatory cytokines in *in vivo* has been shown to protect rodents from insulin resistance thus linking pro-inflammatory mediators to one of the hallmarks in T2D [38], [39]. Further, this pro-inflammatory milieu has been associated with an imbalanced T cell compartment particularly a decreased CD4^+^ Treg compartment traditionally associated with chronic inflammation [40], [41].

Herein, we provide evidence that subjects at elevated risk to develop T2D show a reduction of a subset of T cells and B cells, previously demonstrated to possess immunosuppressive function, with increased FPG and BMI, respectively. [22], [23], [42]. This modulation may be indicative of an early trigger for the chronic inflammation that likely underlies the metabolic disorders of insulin resistance, obesity and T2D.

Our finding, that a reduction of TLR4 expressing B cells showed a significant correlation with increasing BMI, independent of age and sex, is interesting. Tian *et al*. showed that TLR4 expressing B cell have immunosuppressive properties inhibiting Th1 responses and preventing development of T1D [42]. The decrease of TLR4^+^ B cells in the blood may thus relate to exaggerated pro-inflammatory cytokine profile previously observed in patients with elevated BMI [43]. Further, TLR4 expressing T cells have been shown to promote suppressive function of Tregs [22], [23]. Our observation demonstrating a negative correlation between FPG and number of TLR4^+^ CD4^+^ T cells suggests that the reduction of the suppressive TLR4^+^ CD4^+^ T cells might be partially responsible for the exacerbated immune activity noted in the chronically inflamed T2D patients [9]. A similar reduction of the total number of Tregs has previously been shown in T1D patients where it was linked to initiation or acceleration of the disease [44] [45].

Furthermore, we observed a shift from Tregs i.e. CD3^+^CD4^+^CD25^+^CD127^−^ cells towards TLR4^+^ T cells with age demonstrating a potential plasticity in the T cell pool and emphasize that several populations of Tregs need to be evaluated in parallel to ensure better understanding of the complex biology. Previously the frequency of circulating Tregs has been shown to be increased or unchanged with age both in man and in mouse [32], [46], [47]. Raynor *et al*. suggested that the increase of Tregs in elderly could participate in the enhanced frequency of infectious diseases and cancer with age [46]. Our results in this cohort of subjects at elevated risk to develop diabetes suggest that sub-populations of Tregs might develop differently due to the metabolic syndrome. A striking plasticity between the two TGFβ dependent T cells subsets, Tregs and Th17 cells, exists based on additional signals from the environment [48]. With diabetic subjects having progressively elevated levels of TGFβ with disease severity, these patients may be pre-disposed to both polarization programs leaving polarization dependent on the additional environmental or risk factors [49]. Consequently the reduction of the Tregs described herein raises the possibility that certain diabetic risk factors such as obesity may skew the T cell response towards the Th17 phenotype rather than the Treg phenotype [40]. Indeed, the T cells in our cohort showed an inflammatory cytokine signature with detectable levels of inflammatory cytokines in resting T cells which was accentuated rapidly upon re-challenge potentially suggesting that the circulating T cells expressed a pre-activated phenotype. This is in agreement with Stentz *et al*. and M. Jagannathan-Bogdan, who showed elevated cytokine levels in peripheral blood T cells in T2D compared to healthy subjects [40], [50]. Even though the T cells evaluated in our subjects seem to have an activated phenotype based on their cytokine expression we cannot conclude from the observed data if this is a result of these patients being at high risk to develop T2D or if this is a general feature of T cells coming from healthy individuals since we could not observe any correlation between the increased levels of pro-inflammatory cytokines and the increase in metabolic risk factors studied here i.e. the T cells from the four pre-diabetic patients expressed similar amount of pro-inflammatory cytokines as the rest that had normal blood glucose but were classified as being at high risk to develop diabetes. Therefore it would be highly relevant to expand this study to a larger number of type 2 diabetic participants or to make a comparison with a control group i.e. not at risk of developing T2D to address if the inflammatory cytokine signature observed in our T cells is a result of increased risk of developing T2D.

Interestingly, Fabrizi *et al*. recently demonstrated that mRNA IL-21R expression in both obese human subjects and in animal models was increased compared to lean controls [10]. Migration to adipose tissue is dependent on an array of chemokines released from the residual cells as well as from invading leukocytes [11]. This chemokine profile is altered due to signals in the local milieu. Hence our data demonstrating a negative correlation with an increased BMI, but not FPG, of IL-21R expressing T cells is intriguing. This observation proposes a sequential development of adipose inflammation in subjects with high BMI where the systemic IL-21R expressing T cells initially are attracted to the adipose tissue and hence are reduced in the systemic compartment. Once the obese subject starts to show elevated FPG, the chemokine signature in the adipose tissue could be altered potentially due to the elevated TGFβ leading to re-distribution of the IL-21R T cells and thus explaining the absence of negative correlation of IL-21R in the blood with increased FPG.

CPCs have been shown to be reduced in patients with insulin-dependent and non-insulin-dependent diabetes as well as in other chronic diseases like rheumatoid arthritis, chronic renal failure, chronic heart failure and stroke [30], [31]. Evidence has been put forward that the reduced number of CPCs in inflammatory diseases is a result of exhaustion as extensive continuous repair is required [51]. The number of CPCs in our cohort evaluated showed a trend to reduced numbers in the circulation 2 h post meal. This suggests that the CPCs, in these subjects at risk to develop diabetes, might respond quickly to exogenous stimulations such as food intake and hence have an intact migratory capacity. Further the frequency of CPCs both before and after food intake was significantly higher in men than in women. These results emphasize the importance to ensure that analysis of this very small population in clinical studies needs to be conducted in a controlled manner not confounded by food intake and sex that might influence the results obtained. Alteration of blood cell frequencies after food intake has previously been described for various leukocyte populations [28].

## Conclusions

Taken together, our results show that in a small cohort of subjects with elevated risk to develop diabetes a modulation of some peripheral blood immune cell population associated with increase in BMI and FPG occurs. Overall, the profile in these subjects at risk indicates an imbalance of lymphocytes associated with augmenting and controlling the inflammatory metabolic syndrome and hence might provide novel insights to the development of T2D.

## Supporting Information

Figure S1 **Overview of the analysis strategy.** In order to identify four out of 71 highest ranking immune cell markers a random forest analysis was made for each metabolic risk factor as well as for age and sex separately. The four highest ranking markers were further analyzed by linear/logistic regression analysis. All metabolic risk factors were adjusted for age and sex.
(TIF)

Figure S2 **Fasting insulin associations.** FI (pmol/l) vs frequency of CD31^+^CD34^+^CD45^dim^CD133^dim^ CEC (A), number of CD3^+^CD4^+^CD25^+^CD127^−^ T reg cells (B), total white blood cell count (C) and CD19^+^IL21R^−^TLR4^+^ B cells (D). Red dots represent women and blue dots represent men.
(TIF)

Figure S3 **HOMA-B and HOMA-IS associations.** HOMA-B (%) vs number of CD3^+^CD4^+^IL21R^+^TLR4^−^ T cells (A), number IL-17^+^ in resting CD3^+^CD4^+^ T cells (B), frequency of IL-21^+^ in activated CD3+CD4+ (C) and frequency of CD3+CD4^+^IL21R^+^TLR4^−^ (D). HOMA–IS (%) vs frequency of CD31^+^CD34^+^CD45^dim^CD133^dim^ CEC after meal (E), number of IL-4^+^ in resting CD3^+^CD4^+^ T cells (F), frequency of CD3^+^CD4^+^ T cells after meal (G) and frequency of T reg CD3^+^CD4^+^CD25^+^CD127^−^FoxP3^+^ after meal (H). Red dots represent women and blue dots represent men.
(TIF)
