# Supplementary figures and images for: Reduction of Specific Circulating Lymphocyte Populations with Metabolic Risk Factors in Patients at Risk to Develop Type 2 Diabetes
Source: PLoS One. 2014 Sep 25;9(9):e107140. doi: 10.1371/journal.pone.0107140 (PMC4177835; doi:10.1371/journal.pone.0107140)

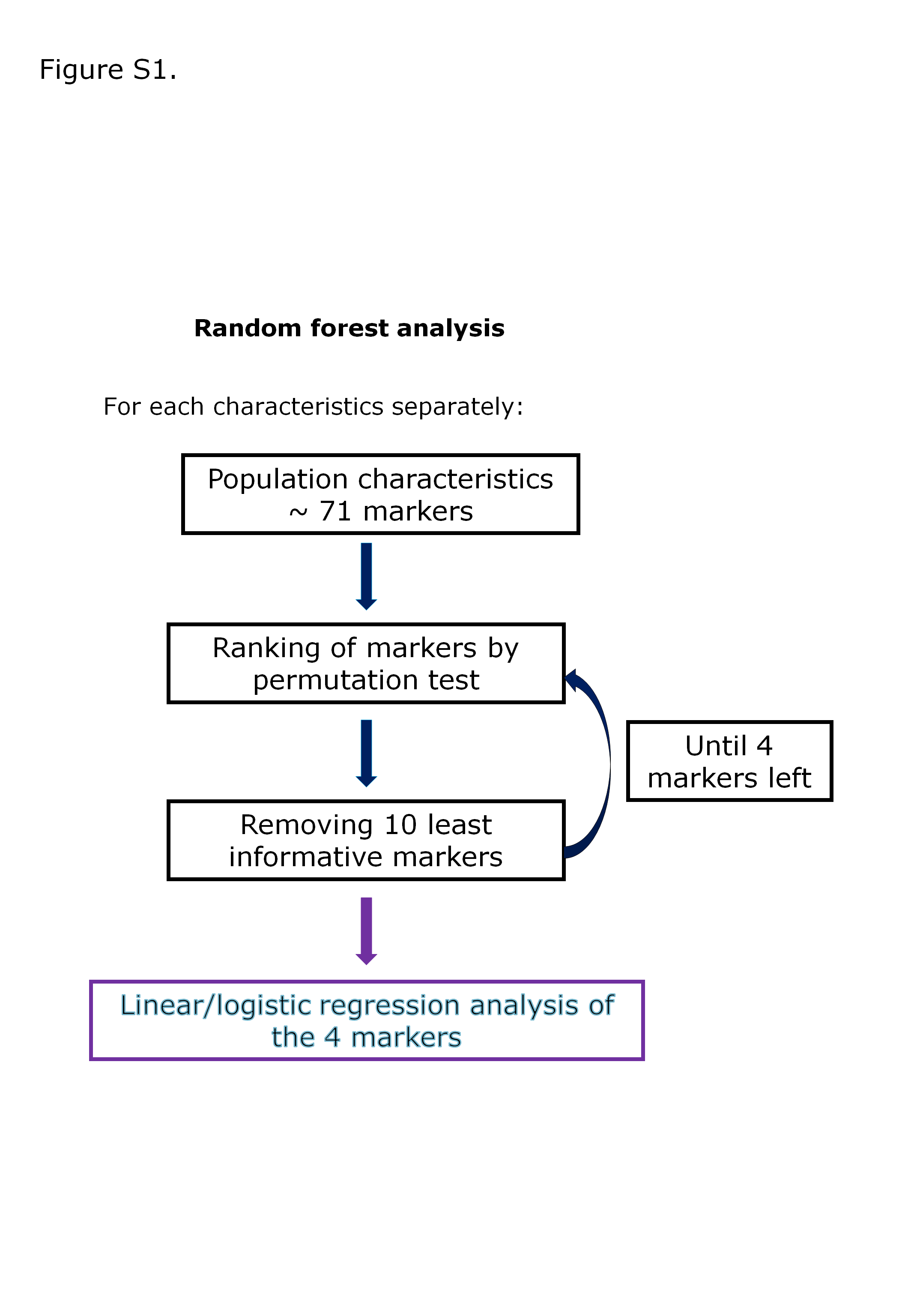

Supplement: Figure S1 — Overview of the analysis strategy. In order to identify four out of 71 highest ranking immune cell markers a random forest analysis was made for each metabolic risk factor as well as for age and sex separately. The four highest ranking markers were further analyzed by linear/logistic regression analysis. All metabolic risk factors were adjusted for age and sex. (TIF) [file pone.0107140.s001.tif]

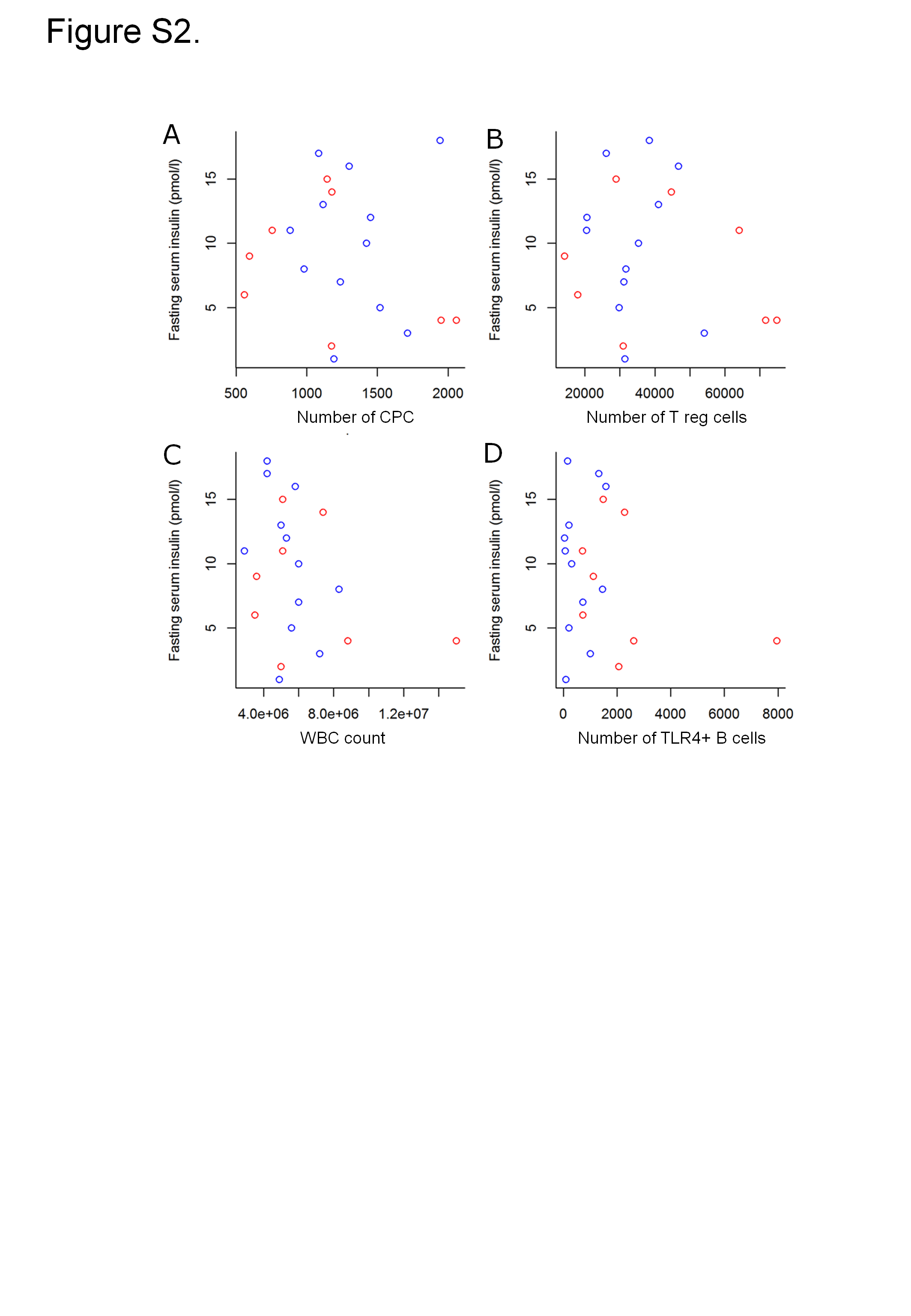

Supplement: Figure S2 — Fasting insulin associations. FI (pmol/l) vs frequency of CD31+CD34+CD45dimCD133dim CEC (A), number of CD3+CD4+CD25+CD127− T reg cells (B), total white blood cell count (C) and CD19+IL21R−TLR4+ B cells (D). Red dots represent women and blue dots represent men. (TIF) [file pone.0107140.s002.tif]

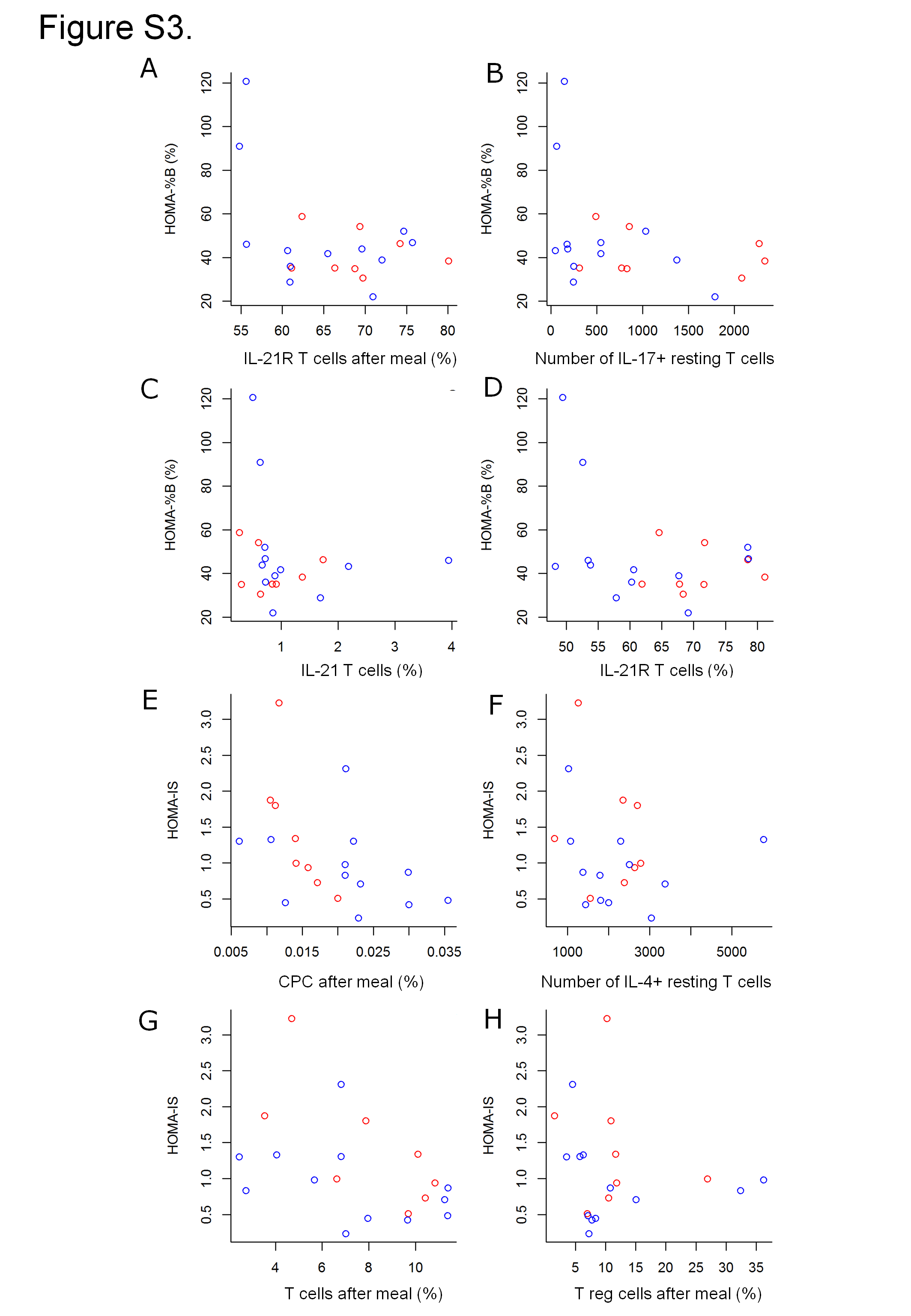

Supplement: Figure S3 — HOMA-B and HOMA-IS associations. HOMA-B (%) vs number of CD3+CD4+IL21R+TLR4− T cells (A), number IL-17+ in resting CD3+CD4+ T cells (B), frequency of IL-21+ in activated CD3+CD4+ (C) and frequency of CD3+CD4+IL21R+TLR4− (D). HOMA–IS (%) vs frequency of CD31+CD34+CD45dimCD133dim CEC after meal (E), number of IL-4+ in resting CD3+CD4+ T cells (F), frequency of CD3+CD4+ T cells after meal (G) and frequency of T reg CD3+CD4+CD25+CD127−FoxP3+ after meal (H). Red dots represent women and blue dots represent men. (TIF) [file pone.0107140.s003.tif]
